# Learning Social Media Content Optimization: How Can SMEs Draw the Users' Attention on Official WeChat Accounts?

**DOI:** 10.3389/fpsyg.2021.783151

**Published:** 2022-01-10

**Authors:** Xueyun Zeng, Xuening Xu, Yenchun Jim Wu

**Affiliations:** ^1^School of Economics and Management, Beijing University of Posts and Telecommunications, Beijing, China; ^2^College of Humanities and Arts, National Taipei University of Education, Taipei, Taiwan; ^3^Graduate Institute of Global Business and Strategy, National Taiwan Normal University, Taipei, Taiwan

**Keywords:** content optimization strategy, psychological need, social media visibility, self-determination theory, SMEs

## Abstract

Application of artificial intelligence is accelerating the digital transformation of enterprises, and digital content optimization is crucial to take the users' attention in social media usage. The purpose of this work is to demonstrate how social media content reaches and impresses more users. Using a sample of 345 articles released by Chinese small and medium-sized enterprises (SMEs) on their official WeChat accounts, we employ the self-determination theory to analyze the effects of content optimization strategies on social media visibility. It is found that articles with enterprise-related information optimized for content related to users' psychological needs (heart-based content optimization, mind-based content optimization, and knowledge-based content optimization) achieved higher visibility than that of sheer enterprise-related information, whereas the enterprise-related information embedded with material incentive (benefits-based content optimization) brings lower visibility. The results confirm the positive effect of psychological needs on the diffusion of enterprise-related information, and provide guidance for SMEs to apply artificial intelligence technology to social media practice.

## Introduction

The emergence of artificial intelligence, blockchain, and big data is bringing infinite possibilities for future changes in business logic, such as information distribution, marketing, customer relationship management, etc. More importantly, artificial intelligence can be superimposed on any other new technology, and its converging use with social media is accelerating the digital transformation of business. A large number of small and medium-sized enterprises (SMEs) are gradually applying artificial intelligence technology for business analysis to spread information and attract the attention of social media users. Especially, social media has been adopted by more and more SMEs for its low cost, ease of use, its capability to reach users, and establish connections with customers. Our work focuses on the content optimization strategy of SMEs by using social media to publish enterprise-related information to explore which type of content is more visible to users, thereby providing some enlightenment for deepening the application of artificial intelligence in social media.

As a new communication option between enterprises and potential or current customers, social media can help SMEs in understanding and responding to customer needs proactively and efficiently (Tajudeen et al., 2018). There is no doubt that the growth of social media users has encouraged SMEs to realize their ambitions by marketing their products, brands, or services more easily and widely (Eggers et al., 2017; Crammond et al., 2018). As a result, social media is considered as a popular and useful tool for SMEs, which can help managers to overcome resource limitations and create competitive advantage (Brink, 2017; Mujahid and Mubarik, 2021).

Although social media applications create competitive advantages for SMEs (Brink, 2017), the lack of knowledge in using social media has been a rough spot (Kraus et al., 2019). Most SMEs cannot make good use of it, and the unattractive content is quite common. In fact, SMEs frequently use social media ignoring the application of content optimization strategy. Enterprises need to stay visible, but how can they catch public attention?

Although a variety of content strategies have been explored, there is few research on content optimization related to users' psychological needs. Existing research related to content strategies includes: social interaction (Gan and Li, 2018), passing time (Malik et al., 2016), information seeking (Gan and Li, 2018; Pant and Pant, 2018), entertainment (Khan, 2017), information usefulness (Rauniar et al., 2014), and so on. However, people's choices are rooted in inner needs, in which psychological needs are seen as essential parts of living entities (Deci, 1975; Deci and Ryan, 1985). Self-determination theory (SDT) provides a theoretical basis for this, including autonomy, relatedness, and competence (Deci, 1975; Deci and Ryan, 1985). It has been widely applied in several fields such as at work (Rathi and Lee, 2017), education (Prestridge, 2019), and sports (Shim et al., 2017). Several studies have contributed to what SDT means in the context of social media, confirming the effects of psychological activities on marketing (Zhang et al., 2014) and content consumption (Kanuri et al., 2018). This implies that the psychological needs may play an important role in drawing more users' attention on social media.

We employ SDT to illustrate the effects of content optimization related with psychological needs (autonomy, relatedness, and competence) on the visibility of SMEs' articles on official WeChat accounts. WeChat is a well-known application in China, and the number of WeChat monthly live users reached 1.15 billion in 2019 (Tencent., 2019). Leveraging on this, WeChat account has become a must-have Internet tool for most enterprises in China. An analytical framework of content optimization strategy related to both psychological needs and material incentives is established, and the visibility of each WeChat article is measured quantitatively with analytic hierarchy process (AHP). The results of regression analysis show us that content optimization based on psychological needs (heart-based content optimization, mind-based content optimization, and knowledge-based content optimization) can significantly increase users' attention, whereas material incentives (benefits-based content optimization) cannot significantly improve visibility. Considering the lack of research on users' psychological need, our work is of significance to the existing literature. This study can help SMEs optimize content strategy and make better use of social media.

This work is structured as follows. In Section Theoretical framework, we describe the theoretical background, where the concept of social media visibility is explained. In Section Hypothesis development, hypotheses are proposed with the intent of figuring out the effects of content optimization strategies. In Section Research method, the research methodology and data sets are presented. Section Results contains the empirical results. Section Discussion discusses the effects of content optimization. Finally, we summarize the main conclusions of the study in Section Conclusion.

## Theoretical Framework

### Social Media Visibility

With the rapid development of Internet, the competition for online visibility is getting increasingly fierce (Drèze and Zufryden, 2004). Existing research emphasized the significance of visibility (Dutot and Bergeron, 2016; Osch and Steinfield, 2018), such as the effects of visibility on web traffic (Drèze and Zufryden, 2004), advertising efficiency and equity value (Fang, 2014). Moreover, social media visibility has been developed for multiple objects, including website visibility (Pant and Pant, 2018), blog visibility (Dennis et al., 2016), and the online visibility from internal employees (Leonardi, 2014; He et al., 2020; Zhou and Mou, 2021). A few preliminary definitions of visibility are given based on specific scenarios. In Drèze and Zufryden (2004), visibility is “the extent to which a user is likely to come across a reference to a company's website in his or her online or of?ine environment.” In Li et al. (2019), the term “social visibility” refers to the visibility of the product's consumption or usage to one's interpersonal networks online or offline. In Yang and Kent (2014), “Visibility refers to the public presence of an individual or organization in the media, and has an influence on organizational perceptions in times of crisis, buying preferences, and trust.” In Shmargad and Watts (2016), the visibility is that one's actions are visible to other users in the media. In Osch and Steinfield (2018), visibility is defined as the relative ease with which, a user could locate relevant information and individuals within the organization.

Focusing on the competition for users' attention, this work defines “social media visibility,” that is, the extent to which the enterprise-related information reaches and impresses users on social media. The term “reach” denotes that what enterprises want to convey are known by users, and “impress” means that contents users received will power them sufficient motivation to respond (i.e., read, share, and comment). In the context of social media, enterprises would like to achieve the dissemination of enterprise-related information in order to gain users' attention and improve their competitiveness and performance. Enterprise-related information includes: (1) products and service; (2) events and participation requirements; (3) core values and culture of enterprises, etc.

### Content Optimization Strategy

Content should be tailored to users' tastes (Dennis et al., 2016) as well as users' needs. Individuals tend to search for information before purchase (Klein, 1998), and so high-value information perform well on social media (Stieglitz and Dang-Xuan, 2013). Available enterprise-related information help users to resolve problems about products and services. Some individuals even take the initiative to follow enterprises' social media accounts to gain the accessibility of enterprise-related information (Muntinga et al., 2011; Rokonuzzaman et al., 2020). It means that enterprise-related information possesses the ability to reach users (Pant and Pant, 2018). Beyond information seeking, users have other motivations for consuming and disseminating content. Kanuri et al. (2018) found that content eliciting high-arousal emotions is more likely to be consumed than other contents. Swani et al. (2017) illustrated the outcome of social media messages based on psychological motivation theory. Hence satisfying users' multidimensional needs may be more effective in the dissemination of enterprise-related information.

Self-determination theory is an appropriate theory to explain the motivational process underlying the relationship between content optimization and social media visibility. Psychological needs are essential parts of living entities in SDT, including autonomy, relatedness, and competence (Deci, 1975; Deci and Ryan, 1985). Autonomy refers to the innate need for self-mastery and self-direction. People's choices of social media content are primarily voluntary (Rauniar et al., 2014), and so their decision must be rooted in underlying intentions. When a user perceives something is aligned with his own thoughts or ideas, there is a mental resonance. The user will think that his opinions are expressed and his autonomy is thus achieved. Therefore, we use mind-based enterprise-related information combined with other content (EIO) to capture users' need for autonomy. Relatedness comprises the need to be related with others and maintain relationships. Individuals perceive the need to share their emotions and support each other. Heart-based EIO can fulfill users' social affective needs. That is, users' inner demand is satisfied when they actively share and can be observed by others, thereby achieving relatedness. Competence denotes the need of one's actions to be effective and masterful. Knowledge-based EIO can improve users' ability and competitiveness. When users read knowledge-based EIO, they tend to identify themselves as capable of action, which is one of “competence.”

These needs give rise to a variety of users' engagement in social media usage (Weiger et al., 2019). After the psychological needs are satisfied, individuals are in a state of enough internal motivation to act (Ryan and Deci, 2000; Swani et al., 2014), such as self-presentation and self-expression. The actions are performed as reading, sharing, and commenting on content released by enterprises. In turn, the enterprise-related information of articles will spread to or impress more users, thereby increasing the visibility of the enterprise.

**Figure 1:**
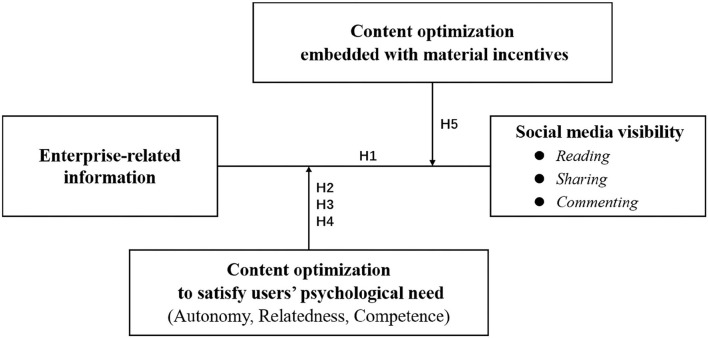
The analytical framework.

We propose the theoretical model of content optimization strategy, as shown in Figure 1. Firstly, Hypothesis 1 is developed to test the visibility of sheer enterprise-related information (SEI) without content optimization. Then, according to SDT, hypotheses 2, 3, and 4 in the combinations of enterprise-related information with heart-based, mind-based, and knowledge-based are proposed, respectively. Lastly, considering that enterprises may also use material incentives to optimize content, hypothesis 5 is developed to explore the effect of material incentives strategy on social media visibility.

## Hypothesis Development

Tapping into account people's desire for information consumption on social media, SMEs try to improve visibility and spread enterprise-related information. However, the visibility of enterprise-related information needs to be enhanced by other content rather than just SEI. The reason is that individuals have other needs besides the information of products and services. The innate needs for relatedness, autonomy, and competence are ubiquitous and critical based on SDT, and so the content related to these is expected to bring a wider range of attention to SMEs. Researchers have agreed that meeting psychological needs can connect with users (Rese, 2006) and retain them (Payne and Frow, 2017). Meanwhile, it has been found that people are willing to seek enterprise-related information for interests, such as lower prices in promotions (Rokonuzzaman et al., 2020). While SEI provides single satisfaction of information acquisition, the enterprise-related information combined with other content (EIO) provides multiple satisfaction of individual needs. We infer that SEI will be weaker than EIO at reaching and impressing users on social media. Hypothesis 1 is as follows.

H1. The visibility achieved by SEI is lower than that achieved by EIO.

Heart-based EIO refers to the content that exerts influence on users' emotions (See Table 1 for specific categorization standards). Previous research has found that relatedness is one of the humans' fundamental psychological needs (Sheldon et al., 2001). According to SDT, individuals have the inclination to relate with others using social media where people exchange with the external world. By commenting and sharing articles on social media, users can express feelings and emotions (Chen et al., 2020). Therefore, enterprises must produce content and express feelings in the users' shoes. For instance, SMEs can post users' normal worries in life and care for them. Such content satisfies users' psychological needs of relatedness (Ryan and Deci, 2000), and so will easily arouse a sense of empathy and being cared among users. Feelings and emotions are motivations for people to transmit content (Swani et al., 2017). Mobilizing users' emotions is an effective way for enterprises to stand out of information distribution and network competition (Rese, 2006). One example is the resonance of novel Coronavirus-related coverage in 2020. It is considered to shock through people's heart, which thus inspires their echo. This can lead to more retweets and stronger impression than posting SEI does. As argued above, we infer that EIO based on heart will promote more visibility than SEI. The following hypothesis is developed.

**Table 1:** The classification criteria of content.

**Category**	**Classification criteria**
SEI	The content exerting influence on users by the attraction of only the enterprise-related information, e.g., 1. Information related to products and service 2. Information related to events and participation requirements 3. Other enterprise information, such as core values of enterprises
Heart-based EIO	The content exerting influence on users through one's heart, e.g.,
	1. Involving inner joy, peace, excitement affection, entertainment, fantasy, escapism, enjoyment and expressing one's emotions
	2. Responding to hot events or attaching key words to celebrities and famous spots and shocking through people's heart or arousing their inner curiosity and emotions
	3. Creating a sense of empathy and giving a sense that the person in the article has something in common with themselves
	4. Tapping provocative or extreme key words, making mutual conflicts or using contradictory words
	5. Using bandwagon effects to create psychological pressure
Mind-based EIO	The content exerting influence on users through one's mind, e.g.,
	1. The way of thinking is rational
	2. Having a good thinking, rigorous, logic or a critical view
	3. Steering users to contemplate themselves, society and the nature
	4. Aiming at igniting reasoned thinking in public, such as attaching hot news to its text or title
Knowledge-based EIO	The content exerting influence on users by the attraction of the knowledge, e.g.,
	1. Satisfying users' long-term search for methods and skills
	2. Providing fragmented but useful knowledge directly
	3. Using tactics that allow a user to obtain embedded resources only when he/she shares the WeChat article to his/her friends circle
Benefits-based EIO	The content exerting influence on users by the attraction of potential benefits, e.g.,
	1. Cash or coupon incentives
	2. Some benefits-based parts, e.g. a purchase link to WeChat shopping mall with a promise of discounts

H2. The visibility achieved by heart-based EIO is larger than that achieved by SEI

Mind-based EIO refers to the content exerting influence on users' thoughts (Table 1 for specific categorization standards). Official WeChat accounts provide large amounts of rational content for users, such as critical view and logical thoughts. Users regard this type of content as material to think and exercise their own way of thinking, and they can select freely what they prefer. It makes users feel free and volitional (Rathi and Lee, 2017) and satisfies their demand for autonomy of thinking. It has been found that users on Facebook are more likely to comment and share the messages with logical information, for which commenting and sharing are cognitively triggered behaviors (Kim and Yang, 2017). This demonstrates that people are inclined to think independently and consume logical content. It is reasonable to believe that WeChat is as the same. In a word, it can be expected that EIO based on the mind has a greater impact on visibility than SEI does. We propose the hypothesis below.

H3. The visibility achieved by mind-based EIO is larger than that achieved by SEI.

Knowledge-based EIO refers to the content exerting influence on users by the attraction of knowledge (Table 1 for specific categorization standards). Individuals have the pursuit of self-actualization and development (Cao et al., 2013). Knowledge could be a resource for self-development. Abundant knowledge meets users' need to learn and deal with the complex and changing world, assisting in the satisfaction for competence (Ryan and Deci, 2000). The gratification of knowledge exists on Facebook (Manasijevi et al., 2016). Likewise, knowledge is desired on WeChat. The content of enterprise-related information combined with knowledge is apt to attract knowledge seekers. Knowledge-based EIO involves fragmented, but useful knowledge for users, often presented directly in SMEs' WeChat articles. Another kind of knowledge-based EIO exists in such a way that users have to share articles to help transmit enterprise-related information in order to exchange for learning resources. With the proliferation of online resources and an increase in accessibility (Prestridge, 2019), the number of users who seek knowledge and resources on WeChat is on the increase. All of this would make knowledge-based EIO more likely to reach and impress users than SEI. The following hypothesis is proposed.

H4. The visibility achieved by knowledge-based EIO is larger than that achieved by SEI.

Benefits-based EIO refers to the content exerting influence on users by the attraction of potential material incentives (Table 1 for specific categorization standards). In addition to psychological needs, potential material incentives can also induce individuals to take action (Ryan and Deci, 2000). Striving for self-interests is human instinct (Muntinga et al., 2011; Cao et al., 2013). As for enterprises, a series of online sales promotion such as discounts, exactly take advantage of people's desire for self-interest (Sheehan et al., 2019). The possibility of earning rewards is an important motivation for internet users (Muntinga et al., 2011), and so content embedded with material incentives presumably fascinates more followers. This kind of content usually incorporates cash or coupon rewards. Some are attached with purchase links to WeChat shopping mall with a promise of discounts. Considering people's benefits-seeking instinct, we think that WeChat users are more likely to read, share, and comment on articles that provide rewards than SEI. The following hypothesis is developed.

H5. The visibility achieved by benefits-based EIO is larger than that achieved by SEI.

## Research Method

### Sample and Data Collection

To test the five hypotheses, we collected the official WeChat account data of 115 SMEs. These SMEs are obtained by random sampling and cover a wide range of industries. Specifically, a list of 300 enterprises registered in Beijing is randomly picked up from TianYanCha (“天眼查”). Then, SMEs are retained according to the classification criteria for large, medium, small, and microenterprises in China. Finally, the 115 SMEs marked with official WeChat accounts are taken as the sample. Three latest articles published from each official WeChat account before September 1, 2019 are tracked for a month, and a total of 345 samples are obtained as the sample. The reason for tracking for a month is that the dissemination of WeChat articles approaches the end after a month. Statistics show that WeChat articles have a life cycle of about 10 days, and after 10 days, the growth rate of daily reading of an article is less than 0.01% (Jing and Wei, 2016). Therefore, it is reasonable to assume that the number of reading, sharing, and commenting of WeChat articles tend to stabilize after a month.

### Measurement of Visibility

We developed a measurement model of social media visibility based on users' subjective perception. In accordance with the definition of “social media visibility” in this work, the model consists of three metrics: reading, sharing, and commenting. Reading can be regarded as the extent to which the content reaches users (Dolan et al., 2016). Sharing shows that an article is shared to one's circle of friends and probably attracts more traffic (Malthouse et al., 2013). Commenting can be viewed as the extent to which the content impresses users, for which users have motivations to express themselves (Shahbaznezhad et al., 2021). These three metrics will be used to measure a comprehensive visibility index that represents the extent to which the content of enterprises released reaches and impresses users on social media.

In this work, AHP is used to measure individuals' perception of reading, sharing, and commenting. It is a method used to estimate the weights of the three subindicators in the visibility measurement model by pairwise comparison. The normalized weights of reading, sharing, and commenting adds up to 1. According to the self-perception theory, people infer their own minds by observing their own behaviors (Dico, 2018). For example, those who observe that more efforts have been paid for commenting than reading will be more impressed from commenting and so will give it a higher rating. Since people are able to clearly perceive the importance represented by each indicator, an AHP-based questionnaire is used to review the efforts people are willing to pay while clicking to read, share, and comment on a WeChat article. This indirectly measures the extent to which enterprise-related information reaches or impresses users.

Before the formal release of our questionnaire, some college students were invited to fill out the questionnaire of the original version and give suggestions to improve its comprehensibility. Then the original version is revised according to participants' feedback and formal questionnaires are conducted among WeChat users. The final questionnaire is available in Appendix A (Appendix Table 1 in Supplementary Material). Using the AHP, we measured the relative weight of reading, sharing, and commenting by 1/9 to 9. Users are invited to rank the importance of reading, sharing, and commenting. For example, a user compared the perceived importance of sharing and reading, that is, the effort it takes for him to click read or share. If sharing is five times as important as reading for him, then the weight of sharing is 5. He also has to give his answer about the perception of the importance of reading as opposed to sharing. If the second answer is logically consistent with the first, such as 1/5, it would be retained. If the second answer contradicts the previous one, such as 3, the answer is eliminated. A total of 237 responses are collected and scrutinized. A valid sample of 196 responses were finally obtained. The demographic information of respondents is consistent with the data set in Gan and Li (2018) and the report released by Tencent. (2016). It can be reckoned as a reliable data set.

Using the data obtained from the questionnaire, a judgment matrix comparing the three indicators is obtained. The eigenvectors are calculated from asymptotic normalization coefficient, namely the relative weights of “Read, Share and Comment” in Table 2.

**Table 2:** Judgement matrix and paired comparisons.

**Matrix**	**Read**	**Share**	**Comment**	**Weight $\bar{\text{w}}$**	**Normalization coefficient**
Read	1	0.4011	0.3991	0.5430	0.16
Share	2.4932	1	0.3702	0.9737	0.29
Comment	2.5054	2.7010	1	1.8915	0.55
Total				3.4081	1.00

*w_Read_ = 0.16, w_2_ = 0.29 w_Share_ = 0.29, w_3_ = 0.61 w_Comment_ = 0.55, CR = 0.094 < 0.1*.

It turns out that commenting requires the most efforts (w_*Comment*_ = 0.55), sharing needs the second (w_*Share*_ = 0.29), and reading needs the least (w_*Read*_ = 0.16). There are several reasons for this result. Firstly, sharing is useful in reaching a larger scope, and it implies the sender's approval for the WeChat article. Thus, “Share” is weighted higher than “Read.” Second, the weight of commenting represents a higher level of mental activity than that of sharing. Sharing does not require content output, and people often repost quickly without comments, so WeChat users generally consider it easier to do. In contrast, commenting requires more effort from users. The time cost, writing cost, and mental thinking all reflect the user's deeper perception of the content. Furthermore, sharing tends to be more frequent than commenting on WeChat. In a word, WeChat users are justified in believing that the perceptual weight of “Comment” is higher than that of “Share” (w_*Comment*_ > w_*Share*_). Finally, the visibility scores of 345 articles selected in this work were obtained according to equation (1).


(1) $$\begin{array}{r} Visibility\;=\;0.16\times Read+0.29\times Share+0.55\times Comment \end{array}$$

### Independent Variables

All WeChat articles in this work were first classified into two sorts, namely SEI and EIO. SEI article contains sheer enterprise-related information. “Sheer_EI” is a dummy variable, equaling 1 when the article only has enterprise-related information without other content. EIO article contains enterprise-related information combined with other contents. “EIO” is also a dummy variable, equaling 1 when the article has both enterprise-related information and other contents. All EIO articles are divided into four categories: heart-based EIO, mind-based EIO, knowledge-based EIO, and benefits-based EIO. The content classification criteria of WeChat articles is shown in Table 1.

To ensure the consistency and credibility of classification, two assistants have several training sessions. Each assistant classifies 345 articles independently. Dennis et al. (2016) focused on the most salient characteristic even though content involves a variety of dimensions. Likewise, we allow one type of content to be classified for each article to catch its most important feature. The interrater reliability is tested with Scott's Pi, which is considered acceptable (Scott's Pi = 0.8994 > 0.80).

### Control Variables

Several control variables related to the propagation of WeChat articles are included to our model. “Modality” is a categorical variable, ranging from 1 to 4, produced to represent the number of media modalities. There are four patterns in WeChat articles: text, audio, picture, and video. Human's perceptual systems combine sensory features from different modalities to yield more reliable stimulus. The effect of multiple modes is thus more profound than that of a single sensory modality. We infer that the more media modalities, the more impressed the users will be.

“Position” is an indicator variable which is equal to 1 if the article is at the first place of several articles. Ghose and Yang (2009) find that conversion rates in search engine result pages are highest at the top and decreased with the page down. Peng et al. (2016) found that publishing position has a positive impact on the amount of reading. Users' attention or memory to the first one is assumed to be greater.

“Time” is an indicator variable equal to 1 if an article is posted during active periods. There are peaks and valleys in social media usage. Kanuri et al. (2018) argue that it is highest in the morning, moderate in the evening, and lowest in the afternoon. Peng et al. (2016) regard 8:00–14:00 and 18:00–20:00 during a day as two active periods for WeChat users and find that posting time significantly affects the number of readings. If the posting time is in these two periods, it is regarded as “publishing” during active periods.

“Followers” are divided into a nine-point scale based on the number of fans, ranging from 1 (less fans) to 9 (more fans). Specifically, “Followers” equal to 1 if the number of fans is less than 10,000. “Followers” equal to 9 if the number of fans is more than 4,800,000. Then we evenly divided the other parts. The more the followers, the stronger the information diffusion force of WeChat articles is. Assuming that the click rate of followers is the same, the account with more followers will achieve higher visibility. Therefore, it is indispensable to control the number of followers.

## Results

Table 3 reports the descriptive statistics of variables in this study. The mean of “ln(Visibility)” is 7.1773, the maximum is 10.0388, and the minimum is 1.1631. The “Sheer_EI” is 21.45% of the total, “Heart” accounts for 31.89%, “Mind” accounts for 19.42%, “Knowledge” accounts for 13.04%, and “Benefit” accounts for 14.20%, respectively. “Modality” conforms to normal distribution, and its mean is close to the median of 2. It indicates that two modalities are commonly used in WeChat articles. “Position” shows that 58.55% of articles come from the first position, and “Time” shows that 47.54% of articles are published at active periods.

**Table 3:** Descriptive statistics for all regression variables in this study.

**Construct**	***N***	**Mean**	**Median**	**SD**	**Min**	**Max**
ln(Visibility)	345	7.1773	7.3909	2.1773	1.1631	10.0388
Sheer_EI	345	0.2145	0	0.4111	0	1
Herat	345	0.3188	0	0.4667	0	1
Mind	345	0.1942	0	0.3962	0	1
Knowledge	345	0.1304	0	0.3373	0	1
Benefit	345	0.1420	0	0.3496	0	1
Modality	345	1.9913	2	0.4210	1	4
Position	345	0.5855	1	0.4933	0	1
Time	345	0.4754	0	0.5001	0	1
Followers	345	4.2087	4	2.2000	1	9

Analysis of intergroup differences in visibility between SEI and EIO is done by Mann–Whitney *U*-test. The results are shown in Table 4. The mean of “ln(Visibility)” achieved by SEI is significantly lower than that of EIO (*p* = 0.000 < 0.01). The average score of “Read, Share and Comment” of SEI is also significantly lower than that of EIO at the level of 1%, respectively. It indicates that SEI is less than EIO at reaching and impressing users. The result is consistent with Hypothesis 1.

**Table 4:** Means of the visibility metrics in both *Sheer_EI* and *EIO*.

	***Sheer_EI***	***EIO***	**Difference**	***P*** **-value**
ln(Visibility)	6.3603	7.4004	1.0401	0.000***
Read	15,373.027	29,148.542	13,775.515	0.000***
Share	85.743	422.616	336.873	0.000***
Comment	14.649	19.273	4.624	0.009***

*74 articles are SEI, while 271 articles belong to EIO*.

*** *p < 0.01*.

The results of the regression analysis of model (1) are shown in Table 5. As expected, under the control of other factors, the coefficient of “Sheer_EI” is found to be significantly negative on “ln(visibility)” (β1 = - 0.574, *p* = 0.000). Hypothesis 1 is supported. “Position” (λ6 = 1.318, *p* = 0.000) and “Followers” (λ8 = 0.812, *p* = 0.000) are found to exert significant impacts on visibility. “Time” shows that it is insignificant whether articles are published in active time periods, namely 8:00–14:00 and 18:00–20:00 (λ7 = 0.124, *p* = 0.324), perhaps readers on WeChat are not time-sensitive.

**Table 5:** Results of the regression analysis of model (1).

**Measure**	**Coefficient**	**Std.Error**	***T*** **-stat**	***P*** **-value**	**VIF**
Constant	2.754	0.336	8.188	0.000***	
Sheer_EI	−0.574	0.154	−3.730	0.000***	1.029
Modality	0.150	0.151	0.991	0.323	1.044
Position	1.318	0.131	10.093	0.000***	1.067
Time	0.124	0.126	0.987	0.324	1.021
Followers	0.812	0.029	28.061	0.000***	1.041
R-Sq(adj) = 0.718					

*** *p < 0.01.*

*ln (Visibility) = α_0_+β_1_Sheer_E_I+β_2_Modality+β_3_Position+β_4_Time+β_5_Follower+ε (1)*.

**Figure 2:**
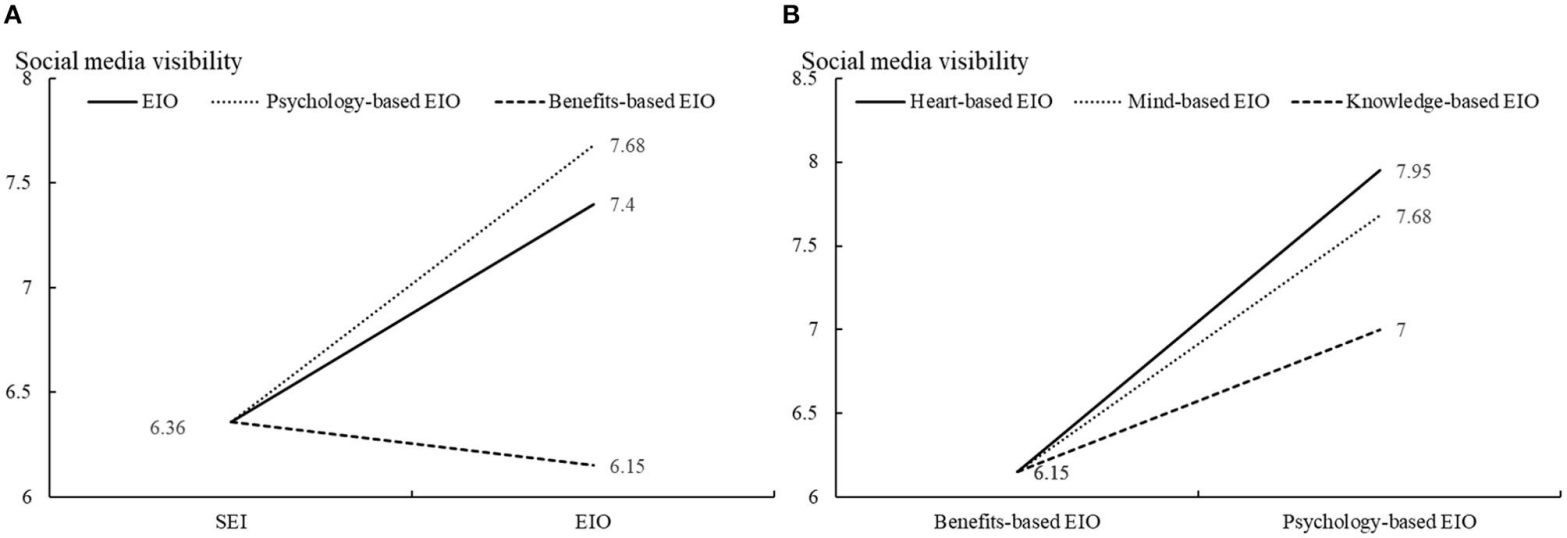
The effect of content optimization strategies on social media visibility. **(A)** The effect of EIO and SEI on social media visibility. **(B)** The effect of psychology-based EIO and benefits-based EIO on social media visibility.

Figure 2 illustrates the effect of different content optimization strategies on visibility. In Figure 2A, the visibility of psychology-based EIO (mean = 7.68) is significantly higher than that of SEI (mean = 6.36, *p*-value = 0.000), but the visibility of benefits-based EIO (mean = 6.15, *p*-value = 0.302) is lower than that of SEI. In Figure 2B, the visibility of psychology-based EIO is significantly higher than that of benefits-based EIO (*p*-value = 0.000). Specifically, heart-based EIO, mind-based EIO, and knowledge-based EIO all have higher visibility than benefits-based EIO. The results are consistent with H1, H2, H3, and H4, and H5 is rejected.

The regression analysis results of model (2)–(5) are shown in Table 6. “Heart × EIO” (λ_1_ = 0.876, *p* = 0.000), “Mind × EIO” (λ_2_ = 0.634, *p* = 0.004), and “Knowledge × EIO” (λ_3_ = 0.627, *p* = 0.008) are major contributors to SMEs' social media visibility. Hypothesis 2, 3, and 4 are supported. The coefficient of “Benefit × EIO” has a significant negative impact on visibility (λ_4_ = −0.752, *p* = 0.000). This is contrary to Hypothesis 5. All variance inflation factors are far less than 2.

**Table 6:** Regression results of content optimization strategies.

**ln(Visibility)**	**Model (2)**	**Model (3)**	**Model (4)**	**Model (5)**
EIO	0.574***	−0.033	−0.028	0.719***
Psychology × EIO		0.752***		
Heart × EIO			0.876***	
Mind × EIO			0.634***	
Knowledge × EIO			0.628***	
Benefit × EIO				−0.752***
Modality	0.150	0.174	0.170	0.174
Position	1.318***	1.238***	1.237***	1.238***
Time	0.124	0.095	0.118	0.095
Followers	0.812***	0.796***	0.793***	0.796***
Constant	2.180***	2.250***	2.260***	2.250***
Observations	345	345	345	345
R-squared	0.722	0.735	0.737	0.735

*** *p < 0.01*.

## Discussion

### Primary Findings

In this study, the first finding is that the visibility achieved by SEI is significantly less than that of EIO (see Tables 4, 5). This means that if a firm disseminates enterprise-related information without content optimization, its visibility can be reduced. To be more precise, EIO related to psychological needs (heart-based EIO, mind-based EIO, and knowledge-based EIO) perform better than SEI in reaching and impressing users (see Figure 2; Table 6). The second finding is that EIO related to material incentives (benefits-based EIO) has a negative effect on visibility. These results are discussed below, respectively.

Heart-based EIO is found to be the dominant contributor to visibility of SMEs. It means that heart-based content optimization is an appropriate choice to impress and reach more users. Relatedness involves the feeling of being connected and cared for (Men and Robinson, 2018). A variety of positive emotions can spur the satisfaction of relatedness, such as joy (Baumeister and Leary, 1995), love, and enjoyment (Sternberg, 1986). The desire for interpersonal attachments can be a fundamental human motivation (Baumeister and Leary, 1995), and so it's extremely common to spontaneously share and comment on heart-based content so as to connect with other users. It is the basic strategy for SMEs to release heart-based content optimization to draw users' attention and maintain close relationships with them.

Mind-based EIO is found to be of significance on social media visibility. This implies that for SMEs, mind-based content optimization can be suitable to establish connections with users and capture their attention. Autonomy is one of the three psychological needs (Ryan and Deci, 2000). In pursuit of the autonomy of thought, individuals use social media to seek content with in-depth thinking that provides resources for making decisions. In this process, people's intuitive and rational thinking can be satisfied. In China, the population of higher education has reached 38.33 million (Ministry of Education of the People's Republic of China., 2019). A variety of people get used to autonomous thinking. Thus, it is easy for mind-based content optimization to capture the public's attention among piles of articles on WeChat.

The knowledge-based content optimization in catching users' attention is also demonstrated. WeChat not only renders people more convenient channels to access learning materials, but also offers enterprises an opportunity to show their expertise in front of the public. SMEs in various industries are capable of providing knowledge and professional skills in specific fields, such as pedagogy, psychology, medicine, nutrition, and environmental science. To gain more attention, SMEs should output clear and specific knowledge. In a word, it is crucial for SMEs to make good use of the knowledge content to establish connections with the target users and spread enterprise-related information.

Benefits-based EIO does not improve social media visibility. It is consistent with Ryan and Deci (2000), who argue that extrinsic rewards undermine intrinsic motivation on social media. Based on Organismic Integration Theory, the degree of self-determination related to external motivation is divided into four types: integration, identification, introjection, and external regulation. These four types of external motivation fall along continuum anchored (Deci and Ryan, 2000). While the internalization degree of “integration” is the highest, the “external regulation” undermines intrinsic motivation (Deci and Ryan, 2000). The reward of WeChat articles is one of such a form of “external regulation.” WeChat posts often give users trivial benefits, which are no longer necessities for people nowadays. Furthermore, users need to perform multiple complex steps, and even input personal information. The cumbersome procedures and trivial rewards often lead to user aversion, which may be the cause of low visibility. People are more likely to be driven by psychological needs than material incentives (Deci and Ryan, 1985; Ryan and Deci, 2000). Users usually consume high-value content (Stieglitz and Dang-Xuan, 2013) instead of rewarding content similar to commercial advertisement, which is not attractive to users.

### Theoretical Contributions

This study contributes to the existing literature in several ways. Firstly, this study extends SDT to social media context. It is of significance to literature on the social media content optimization. Previous research mainly focused on user's gratifications such as entertainment, enjoyment, and utilitarian needs (Rauniar et al., 2014; Malik et al., 2016; Khan, 2017; Gan and Li, 2018). Content optimization on users' psychological needs has been paid little attention to. In this regard, this work explores the role of psychological needs (i.e., autonomy, relatedness, and competence) in the diffusion of enterprise-related information.

Secondly, it is conducive to social media visibility research. Visibility on social networks has been discussed largely in literature, but is mostly qualitative research (Yang and Kent, 2014; Osch and Steinfield, 2018). For this, we extracted the basic elements for conceptualization. A precise definition and a set of quantitative index system for social media visibility are given in this study. The score of visibility is calculated with the weights of reading, sharing, and commenting on WeChat by AHP. It not only provides a visibility index system for WeChat articles, but also can be extended to other social media.

Thirdly, the novelty of this work is rooted in the challenges of SMEs. The existing research mainly focused on knowledge management (Scuotto et al., 2017), marketing strategy (Kraus et al., 2019), and internal communication (Zilber et al., 2019), which are usually extended from research on large companies. This study originates from the current challenges of SMEs, rather than following the research on large enterprises.

### Practical Implications

From our findings, it is crucial that enterprises take the initiative to learn content optimization strategies to satisfy users' psychological needs so as to get more attention. SMEs should make content that meet users' psychological needs to enhance visibility of enterprise-related information. Since emotion is one of the most powerful contributors to human behaviors (Sailunaz and Alhajj, 2019), it would be easy to make people click to read, share, and comment when managers adopt above content optimization strategies to care about users' psychological needs, such as releasing more heart-based EIO in official WeChat accounts. Furthermore, SMEs should avoid relying on material incentives to grab users' attention. The reason is that WeChat articles containing both incentives and business-related content are similar to advertisements, which may turn users off. These content optimization strategies also offer lessons for large companies. Most of them do not have a mature strategy for social media activities (Zembik, 2018), even with adequate resources (Kraus et al., 2019).

### Future Research

Although the content optimization strategy we extracted in this study provides some key factors for this issue, content strategies are complex and difficult to measure in practice. Therefore, some deep learning algorithms, such as SGD, RMSProp, Adam and so on, can develop more complex estimation models and improve prediction accuracy through big data training. It will be able to provide more concrete suggestions to SMEs on content optimization strategy.

## Conclusion

Nearly all contemporary people's behavior is on mobile terminals, which makes social media content optimization crucial. Previous research mainly focused on users' gratifications, and they do not pay enough attention to the information dissemination related to users' inner needs. This work highlights the importance of psychological needs based on SDT and draws the following two conclusions. Firstly, this study confirms to us how content optimization related to users' psychological needs (heart-based content optimization, mind-based content optimization, and knowledge-based content optimization) attracts users to read, share, and comment. Secondly, content optimization based on material incentives fails to improve social media visibility. The reason is that many people have an emotional aversion to the tedious procedures and trivial benefits. These conclusions provide a new starting point for content research, which can be further deepened and expanded in the future.

## Data Availability Statement

The original contributions presented in the study are included in the article/Supplementary Material, further inquiries can be directed to the corresponding authors.

## Author Contributions

XZ: conceptualization, data analysis, writing, review, editing and supervision, and funding acquisition. XX: data analysis, data collection, writing, and review and editing. YW: logic, review, and supervision. All authors contributed to this manuscript and approved the submitted version for publication.

## Funding

This work was supported by the National Natural Science Foundation of China under Grant No.71872020, the Corporate Finance and Innovation Development Research Center in BUPT, Ministry of Science and Technology, Taiwan (109-2511-H-003−049 -MY3).

## Conflict of Interest

The authors declare that the research was conducted in the absence of any commercial or financial relationships that could be construed as a potential conflict of interest.

## Publisher's Note

All claims expressed in this article are solely those of the authors and do not necessarily represent those of their affiliated organizations, or those of the publisher, the editors and the reviewers. Any product that may be evaluated in this article, or claim that may be made by its manufacturer, is not guaranteed or endorsed by the publisher.
