# Supplementary material for: Learning Social Media Content Optimization: How Can SMEs Draw the Users' Attention on Official WeChat Accounts?
Source: Front Psychol. 2022 Jan 10;12:783151. doi: 10.3389/fpsyg.2021.783151 (PMC8791077; doi:10.3389/fpsyg.2021.783151)
Supplement: Supplementary file 1 [file Data_Sheet_1.pdf]

Supplementary Material

Appendix A. Final Questionnaire

Have you ever estimated the number of times you share and comment while reading WeChat articles? In your mind, how many times is reading more important than commenting? Please answer the questions about the importance of reading, sharing and commenting below, and give your opinions on the perceptual weight between each pair of them.

Table A.1 Interpretation of comparative perceptual weight

Value	Interpretation
1	The same importance
3	Slightly more important than the other
5	More important than the other
7	Much more important than the other
9	More important than the other to the extreme
2/4/6/8	The moderate degree of the two adjacent judgments above

Questions:

1. What's your gender?

2. What's your age?

3. How frequent do you use WeChat in a day?

4. How frequent do you read WeChat articles in a day?

5. What do you think of the definition of visibility that "the extent to which the enterprise-related information reach and impress users"?

6. What do you think of the idea that "I'll read the article first and share it later"?

7. What do you think of the idea that "I'll share the article for the title of the article"?

8. In your mind, how many times is the importance of sharing relative to reading in a WeChat article? (assuming the importance of reading is 1)

9. What do you think of the idea that "I'll read the article first and comment it later"?

10. In your mind, how many times is the importance of commenting relative to reading in a WeChat article? (assuming the importance of reading is 1)

11. In your mind, how many times is the importance of commenting relative to sharing in a WeChat article? (assuming the importance of sharing is 1)
